# The ESF Programme on ‘Integrated Approaches to Functional Genomics’. Workshop on ‘Ontology for Biology’

**DOI:** 10.1002/cfg.252

**Published:** 2003-02

**Authors:** Luca Bernardi, Isabel Rojas, Paul van der Vet

**Affiliations:** 1 European Media Laboratory Villa Bosch Heidelberg Germany; 2 Centre for Telematics and Information Technology University of Twente The Netherlands


**Comparative and Functional Genomics**
*Comp Funct Genom* 2003; **4**: 68–70.
Published online in Wiley InterScience (www.interscience.wiley.com). **DOI:** 10.1002/cfg.252

**Feature**

### Conference Report: The ESF programme on 'Integrated Approaches to Functional Genomics'. Workshop on 'Ontology for Biology'

7–8 November 2002, European Media Laboratory, Villa Bosch, Heidelberg, Germany

Luca Bernardi,[1]* Isabel Rojas[1] and Paul van der Vet[2]
[1] *European Media Laboratory, Villa Bosch, Heidelberg, Germany*
[2] *Centre for Telematics and Information Technology, University of Twente, The Netherlands*

**Correspondence to:*
*Luca Bernardi, European Media Laboratory, Villa Bosch, Heidelberg, Germany.*
*E-mail:*
*luca.bernardi@eml.villa-bosch.de*

Received: 2 December 2002
Revised: 4 December 2002

### Introduction

Ontologies are finding increasing use in all kinds of computer applications, and applications in biology are no exception. The main objective of this workshop was to bring together scientists of various disciplines, such as biologists, computer scientists, philosophers and computer linguists, who are working on, or are interested in, the development of ontologies in biology and related fields. We wanted to share experiences, methods, ontologies and tools. The invitation to the speakers mentioned four main questions to focus the discussion:

1. *Why do we need ontologies?* Are ontologies essential for consistent annotation and indexing? Are they essential for automated processing of biological data?
2. *How are ontologies built?* The teams that build ontologies may comprise domain experts, computer scientists and even philosophers in any combination. What are the experiences in building ontologies? In particular, are there working practices that can be shared with other groups?
3. *How are ontologies checked for quality?* What criteria are relevant? Which tools can be used?
4. *How are ontologies used?* It is known that ontologies are used for very diverse purposes, and therefore heterogeneity in ontologies is only to be expected. Additionally, there is the opportunity to draw attention to novel, unexpected purposes.

### The talks

Although the original intent was to organize the workshop as four sessions, each devoted to one of the questions above, speakers tended to address several questions in their talks. We will summarize all talks in an order that, for reasons of exposition, does not reflect the actual order at the workshop. In this Issue, there are seven reviews and

Copyright © European Science Foundation. Published in 2003 by John Wiley & Sons, Ltd.

one paper from speakers at the workshop, and there is also a review by Alexa McCray (National Library of Medicine, USA), who was invited as a speaker but was unable to attend. The workshop offered the opportunity of presenting posters. A total of 10 posters presented an interesting complement to the series of talks. Abstracts of all talks and posters, and slides of the talks, can be accessed via the web page of the workshop at **http://projects.eml.org/sdbv/events/bioontology/**. The slides also point to the webpages of the authors and/or projects.

Two talks were dedicated to the Gene Ontology (GO) project. **Midori Harris [European Bioinformatics Institute (EBI)]** gave an introduction to GO, which is arguably the most prominent example of a biological ontology in the field of functional genomics. She described the classification aspects considered in GO, highlighting its scope, the type of relations considered and its use, mentioning some of the tools that use GO. GO is designed primarily as a tool for humans: to achieve consistent annotations of data in databases, and as an indexing aid. **Rolf Apweiler (EBI)** demonstrated one particular use of GO, the Gene Ontology Annotation project (GOA), which aims at annotating gene products using GO terms in a number of EBI databases, such as SWISS-PROT + TrEMBL and InterPro. In the old SWISS-PROT data model, an annotation and its source are mentioned in different fields, so that their relation is broken. GOA reunites annotation and source.

Although GO is primarily intended to be a tool for humans, computer projects involving, for example, automated text mining, obviously profit from consistent annotation. In this sense, GO prepares for more automated data processing. However, GO is not intended for automated reasoning. The annotations produced in the GOA project will be regarded by computer scientists as knowledge representation, given that they classify and characterize the entries in the annotated databases. This 'knowledge representation language' is less expressive than several other knowledge representation languages. Typically, the less expressive a knowledge representation language is, the less powerful are the capabilities for automated reasoning. Systems in which the reasoning is partly or wholly automated therefore need a more expressive and more formal ontology. Enriching the formal semantic content of GO is one of the goals of the GONG initiative (**http://gong.man.ac.uk**). **Jennifer Williams (Ontology Works, USA)** presented an initial work that also moves in this direction. She enriches GO with formalized background knowledge and formalizes the ontology in other ways. The intention is to use the result as a starting point for systems that are able to reason about biological data.

**Udo Hahn (University of Freiburg, Germany)** and **Alfonso Valencia (Spanish National Center of Biotechnology)** talked about their experiences in using ontologies in systems that perform information extraction from text. Hahn presented his work on the partly automatic extraction of an ontology from the UMLS (Unified Medical Language System) thesaurus. Valencia presented his work on the automatic generation of classifications of gene-product functions using bibliographic information.

With respect to methodologies for the construction of ontologies, **Aldo Gangemi (Italian National Research Council)** presented a series of high-level conceptual tools for building domain ontologies, ontologies for biomedical domains among them. He introduced DOLCE, a foundational ontology containing an axiomatic characterization of basic, domain-independent concepts and relations. He also introduced the ONIONS methodology for the transformation of terminologies into ontologies.

**Steffen Schulze-Kremer (RZPD; Resource Center/Primary Database, Germany)** presented his experience in the development of ontologies for biology. He characterized ontologies in biology and bioinformatics and described the methodology and tools he uses.

These talks were well complemented by those of **Esther Ratsch (European Media Laboratory, Germany)** and **Alain Viari (INRIA Rhône-Alpes)**. Ratsch presented work on the creation of an ontology for the domain of protein interactions. The ontology is developed by an interdisciplinary group that comprises researchers in biology, computer science and computer linguistics. Viari introduced Genostar, a software platform for genomic data integration and analysis. Genostar is based on an ontology of the 'genomic world', represented as a large network of biological entities and their relationships. Viari also presented the work of **Anne Morgat (INRIA Rhône-Alpes)** — who unfortunately was not able to attend — on the Panoramix project. Panoramix aims at federating knowledge

Copyright © European Science Foundation. Published in 2003 by John Wiley & Sons, Ltd. 

bases in the fields of relational annotation of microbial genomes. The system is based on a formal and explicit representation of the biological entities involved in genome analysis.

**Steffen Staab (University of Karlsruhe, Germany)** discussed the set of tools, languages and services that are collectively known as the 'semantic web'. The semantic web aims at interoperable web services. The semantic web is designed to rely on many, decentralized ontologies that have been made available by their owners, rather than on centralized, monolithic ontologies.

**Daniel von Wachter (University of Leipzig, Germany)** offered a more philosophical touch to the workshop. He demonstrated how philosophical viewpoints influence the building of ontologies by means of an example from his own work, which deals with a theory of causality and an ontology of a part of the medical domain. The claim is that by using that theory, the construction of the domain ontology is facilitated.

### Conclusions and future aims

The workshop was the theatre of many discussions, both after talks and in the long intervals between sessions. They reflected how the field is far from established and that even terminological issues play a role. The differences between perspectives (use, objective and even definition of ontology) are closely correlated with the purpose to which the ontology is put. Although this is in itself not a surprising conclusion, it pays to emphasize it because just mentioning the term 'ontology' still suffices to generate a heated debate. Mutual misunderstanding stands in the way of interdisciplinary work, and all agreed that the functional genomics research program is only feasible if researchers from a number of disciplines cooperate. Researchers from different backgrounds should then come to terms with each other, recognizing different use contexts and needs, and different ways to approach the subject. Biological subject matter is quite foreign to computer scientists. Computer scientists cannot reasonably expect biologists to be aware of, or even interested in, how particular applications are built. For a biologist, a computer application is just a tool. It should fulfil the requirements every tool has to fulfil: ease of use, transparency, efficiency and effectiveness. Bioinformaticians fall midway between these two professions. They also approach computer applications as tools but they put their tools to very advanced uses. Bioinformaticians tend to build their software partly, or wholly, themselves, and they can thus function as two-way interpreters.

On the second day, there was a discussion about a possible sequel to this workshop. Interdisciplinary cooperation is best practised in concrete projects, where the benefits of cooperation are visible to all from the outset. It was therefore proposed to organize a hands-on, summer school-like event where a well-defined biological ontology topic is addressed in such a way that biologists, bioinformaticians and computer scientists are all involved. One of the key issues is to define the end-user role for the deliverable of this event, because the end user is the ultimate arbiter on system functionality. These thoughts have to mature before they can be communicated to the community.

### Acknowledgements

The workshop committee would like to thank the European Science Foundation for its generous grant and support. We would also like to thank the other sponsors of the workshop: the Klaus Tschira Foundation (KTS), the European Media Laboratory (EML) and Ace Bioscience. The organizational support of the University of Twente is gratefully acknowledged. Special thanks go to Annette Martin, for her constant and kind support, and her patience in answering our questions regarding the workshop organization. The organizers would like to acknowledge the help of the following persons from the EML and KTS: Andreas Reuter, Bärbel Mack, Kornelia Gorisch, Silke Peters, Peter Thoma, Ursula Kummer, Holger Buckel, Peter Saueressig, Reinhold Weinmann and Alexandra Martin. Last, but not least, thanks to the reviewers and of course to the participants, who made the workshop a very interesting one.

Copyright © European Science Foundation. Published in 2003 by John Wiley & Sons, Ltd. 

